# Different Effects of *Bacillus thuringiensis* Toxin Cry1Ab on Midgut Cell Transmembrane Potential of *Mythimna separata* and *Agrotis ipsilon* Larvae

**DOI:** 10.3390/toxins7124894

**Published:** 2015-12-15

**Authors:** Yingying Wang, Zhaonong Hu, Wenjun Wu

**Affiliations:** 1 Institute of Pesticide Science, College of Plant Protection, Northwest A & F University, Yangling 712100, Shaanxi, China; wang553135575@163.com; 2 Key Laboratory of Botanical Pesticide R & D in Shaanxi Province, Yangling 712100, Shaanxi, China; wuwenjun@nwsuaf.edu.cn

**Keywords:** *Bacillus thuringiensis*, Cry1Ab, *Mythimna separata*, *Agrotis ipsilon*, apical membrane potential, sensitivity

## Abstract

*Bacillus thuringiensis* (*Bt*) Cry toxins from the Cry1A family demonstrate significantly different toxicities against members of the family Noctuidae for unknown reasons. In this study, membrane potential was measured and analyzed in freshly isolated midgut samples from *Mythimna separata* and *Agrotis ipsilon* larvae under oral administration and *in vitro* incubation with *Bt* toxin Cry1Ab to elucidate the mechanism of action for further control of these pests. Bioassay results showed that the larvae of *M. separata* achieved a LD_50_ of 258.84 ng/larva at 24 h after ingestion; *M. separata* larvae were at least eightfold more sensitive than *A. ipsilon* larvae to Cry1Ab. Force-feeding showed that the observed midgut apical-membrane potential (*V_am_*) of *M. separata* larvae was significantly depolarized from −82.9 ± 6.6 mV to −19.9 ± 7.2 mV at 8 h after ingestion of 1 μg activated Cry1Ab, whereas no obvious changes were detected in *A. ipsilon* larvae with dosage of 5 μg Cry1Ab. The activated Cry1Ab caused a distinct concentration-dependent depolarization of the apical membrane; *V_am_* was reduced by 50% after 14.7 ± 0.2, 9.8 ± 0.4, and 7.6 ± 0.6 min of treatment with 1, 5, and 10 μg/mL Cry1Ab, respectively. Cry1Ab showed a minimal effect on *A. ipsilon* larvae even at 20 μg/mL, and *V_am_* decreased by 26.3% ± 2.3% after 15 min. The concentrations of Cry1Ab displayed no significant effect on the basolateral side of the epithelium. The *V_am_* of *A. ipsilon* (−33.19 ± 6.29 mV, *n* = 51) was only half that of *M. separata* (−80.94 ± 6.95 mV, *n* = 75). The different degrees of sensitivity to Cry1Ab were speculatively associated with various habits, as well as the diverse physiological or biochemical characteristics of the midgut cell membranes.

## 1. Introduction

*Bacillus thuringiensis* (*Bt*) Cry toxins are the active ingredients in most widely used biological insecticides and insect-resistant transgenic crops; however, pests can develop resistance, thereby reducing the effectiveness of these toxins [1,2,3]. The narrow spectrum of activity for specific *Bt* toxins also limits the efficacy and causes additional chemical pesticide applications for adequate pest control when they are not controlled or inadequately controlled by the *Bt* toxin gene in transgenic crops [4].

The oriental armyworm *Mythimna separata* Walker (Lepidoptera: Noctuidae) is a severe pest consuming the foliage of cereal crops, particularly wheat, maize, and rice, throughout eastern China [5]. This species belongs to a group of insects readily susceptible to Cry1A toxins [6,7]. The black cutworm *Agrotis ipsilon* Hufnagel (Lepidoptera: Noctuidae), whose larvae live in the top layer of the ground and forage at night, is a major agricultural pest worldwide. The larvae of *A. ipsilon* feed on almost all varieties of vegetables and many important grains by cutting down and partly eating garden and crop plants, especially seedlings [8,9]. Additionally, *A. ipsilon* is described to be a group of more *Bt*-tolerant insects, which are difficult to control with sprayable *Bt* biopesticides or transgenic *Bt* plants [1,10]. The significantly different toxicity of *Bt* toxins from the Cry1A family against the family Noctuidae remains unclear. A generally accepted mechanism for Cry toxins is characterized by the sequential steps of protoxin activation, specific binding, and cell toxicity [11,12]. Crystal proteins are first ingested as protoxins, which are solubilized and proteolytically converted into smaller and protease-stable polypeptides in the insect midgut. These activated toxins bind to specific receptors on the surface of midgut epithelial cells, thereby allowing them to enter the membrane and form poorly selective pores permeable to small molecules, such as inorganic ions, amino acids, and sugars [13,14]. The presence of such pores in the plasma membrane interferes with the cell physiology by disrupting transmembrane ionic gradients, potentially leading to the colloid-osmotic lysis of cells because of the massive influx of solutes from the midgut lumen [11,15]. Consequently, cell destruction extensively damages the midgut epithelial tissue and causes death of the intoxicated larvae. An alternative model proposed the activation of intracellular signaling pathways by toxin monomers binding to cadherin without the need of the toxin oligomerization step to cause cell death [16]. Midgut lesions caused by the toxins lead to septicemia induced by the midgut bacteria, eventually causing insect death [17]. The physiology of the larval midgut epithelium of lepidopterans is characterized by a strong active transport of K^+^ from the hemolymph to the lumen [18,19]. This activity, which is generally thought to be mediated by a vacuolar-type H^+^-ATPase coupled with an electrogenic K^+^/H^+^ antiporter [20,21,22], maintains the large potential difference across the epithelium.

A simple technique for intracellular recording with a standard microelectrode was developed to measure the electrical membrane potential of epithelial cells in freshly isolated lepidopteran larval midgut samples by Peyronnet *et al.* [23]. The recording technique was successfully conducted to investigate the electrical membrane potential of epithelial cells in freshly isolated lepidopteran larval midguts. The ability of different *Bt* toxins to depolarize the apical membranes of gypsy moth (*Lymantria dispar*), silkworm (*Bombyx mori*), and tobacco hornworm (*Manduca sexta*) larval midguts was compared with their *in vivo* toxicities to these larvae [23,24,25,26]. In the present study, the same technique was adopted to measure the membrane potential in freshly isolated midgut samples from *M. separata* and *A. ipsilon* larvae under oral administration and *in vitro* incubation with the *Bt* toxin Cry1Ab to elucidate the mechanism of action and verify whether the membrane potential depolarization is correlated with the different susceptibilities to *Bt* toxins of both larvae for further control of these pests.

## 2. Results and Discussion

### 2.1. Toxicity of Cry1Ab against M. separata and A. ipsilon Fourth-Instar Larvae

Bioassay results demonstrated that *M. separata* was more susceptible to Cry1Ab than *A. ipsilon*. The LD_50_ value of Cry1Ab against the fourth-instar larvae of *M. separata* was 258.84 ng/larva, and the 95% confidence limits for concentration were 208.84–320.81 ng/larva. Moreover, *A. ipsilon* larvae were still alive after ingesting Cry1Ab at 2000 ng/larva after 24 h. Cry1Ab showed no insecticidal activity on *A. ipsilon*. Therefore, *A. ipsilon* is at least eightfold more tolerant to Cry1Ab than *M. separata*.

### 2.2. Midgut Transmembrane Potential of M. separata and A. ipsilon Larvae

The initial apical membrane potential (*V_am_*) and the initial basolateral membrane potential (*V_bm_*) of normal larvae of *M. separata* and *A. ipsilon* are listed in Table 1. Previous studies indicate that impalement is successful when the initial membrane potential is −20 mV or lower [23,27,28]. In the current work, the midgut samples were rinsed with 3 mL of 32K bath solution, and the membrane potential was stable over 5 min. Afterward, 0.4 mL of the 32K solution was extracted, and an equal volume of the 32K solution was directly added to the bath. However, no significant changes of *V_am_* and *V_bm_* were observed after 30 and 20 min, respectively. We only analyzed the recording data of this stable period in the subsequent toxicological experiments.

**Table 1:** Midgut transmembrane potential of *M. separata* and *A. ipsilon* larvae.

Insect	Potential Type	Mean ± SEM/mV	Range/mV	Number
*M. separata*	*V_am_*	−80.94 ± 6.95	−60 to −100	75
	*V_bm_*	−29.47 ± 3.46	−20 to −40	44
*A. ipsilon*	*V_am_*	−33.19 ± 6.29	−20 to −65	51
	*V_bm_*	−29.04 ± 4.54	−20 to −40	47

### 2.3. Effects of Orally Administered Cry1Ab on Midgut Transmembrane Potential

**Figure 1:**
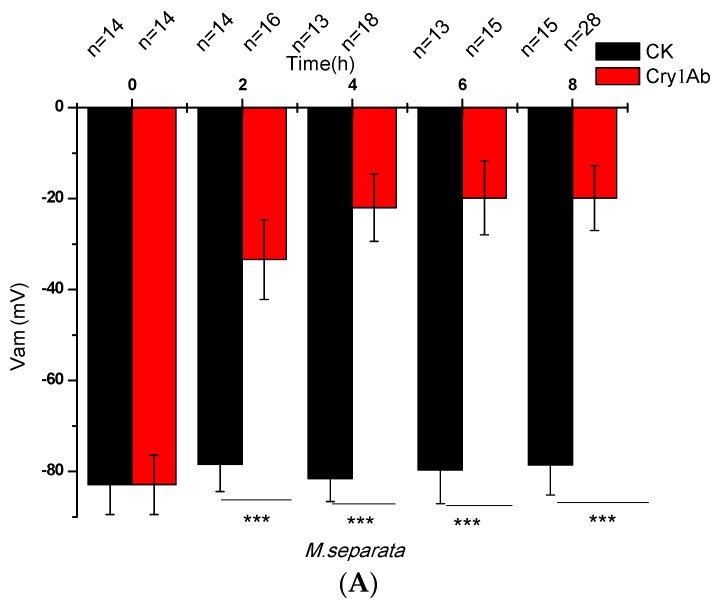

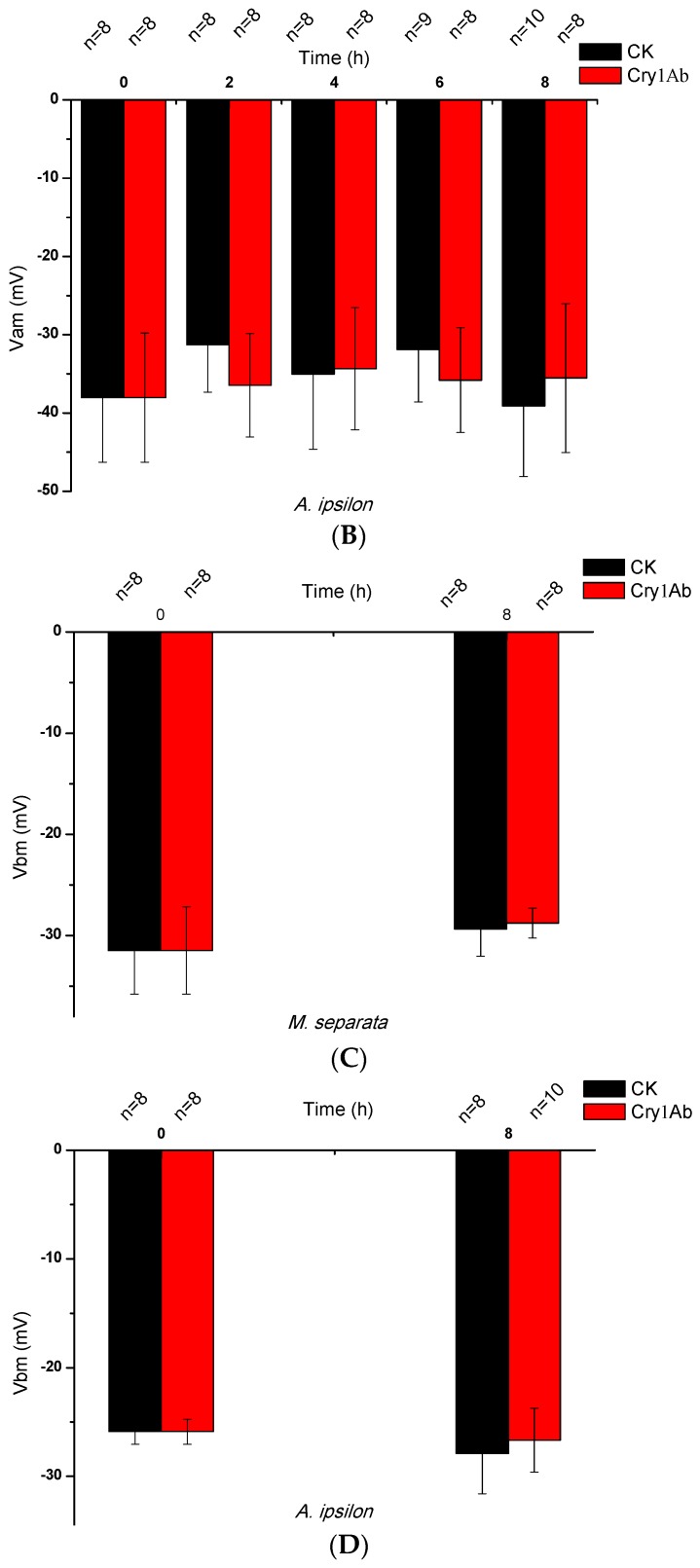
Effects of orally administered Cry1Ab on the midgut epithelial cells of *M. separata* and *A. ipsilon*. The sixth-instar larvae of *M. separata* and *A. ipsilon* were fed with 1 μL of the 32K solution containing 1 and 5 μg of activated Cry1Ab, respectively, and *V_am_* (**A**,**B**) and *V_bm_* (**C**,**D**) were recorded with time. CK means the control which only fed with 1 μL of the 32K solution without toxin. *V_am_* and *V_bm_* are the mean membrane potentials when these values remained stable over 5 min. Values are presented as mean ± SEM for 8 to 28 independent experiments. Note: *** indicates *p* ≤ 0.001.

The *V_am_* was measured directly with time in the sixth-instar larvae of *M. separata* after ingestion of 1 μL of the 32K solution containing 1 μg of activated Cry1Ab. Figure 1A shows the effects of orally administered Cry1Ab on *V_am_* of *M. separata* larvae. The *V_am_* was −82.9 ± 6.6 mV (*n* = 14) and stable for up to 8 h in larvae fed with the 32K solution alone (Figure 1A). The *V_am_* rapidly decreased for those fed with activated Cry1Ab. The *V_am_* dropped to −33.4 ± 8.5 mV (*n* = 16), −22.0 ± 7.4 mV (*n* = 18), −19.8 ± 8.1 mV (*n* = 15), and −19.9 ± 7.2 mV (*n* = 28) after 2, 4, 6, and 8 h, correspondingly, thereby showing that depolarization was time-dependent. The *V_am_* of *A. ipsilon* larvae was not significantly affected by doses as high as 5 μg of activated Cry1Ab (Figure 1B), which coincided with the bioassay result indicating that *A. ipsilon* larvae are insensitive to Cry1Ab. As shown in Figure 1C,D, Cry1Ab did not significantly affect the *V_bm_* of both larvae.

### 2.4. Effects of Cry1Ab Incubated with Midgut Cells on Transmembrane Potential

**Figure 2:**
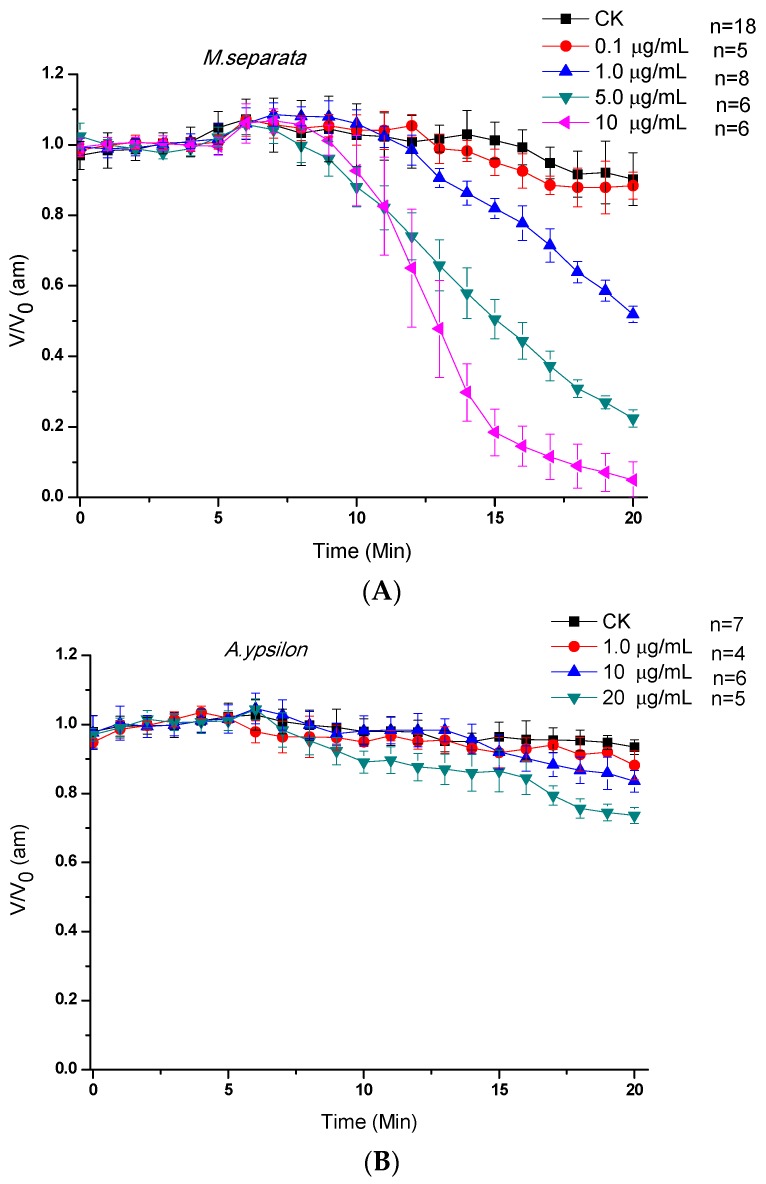

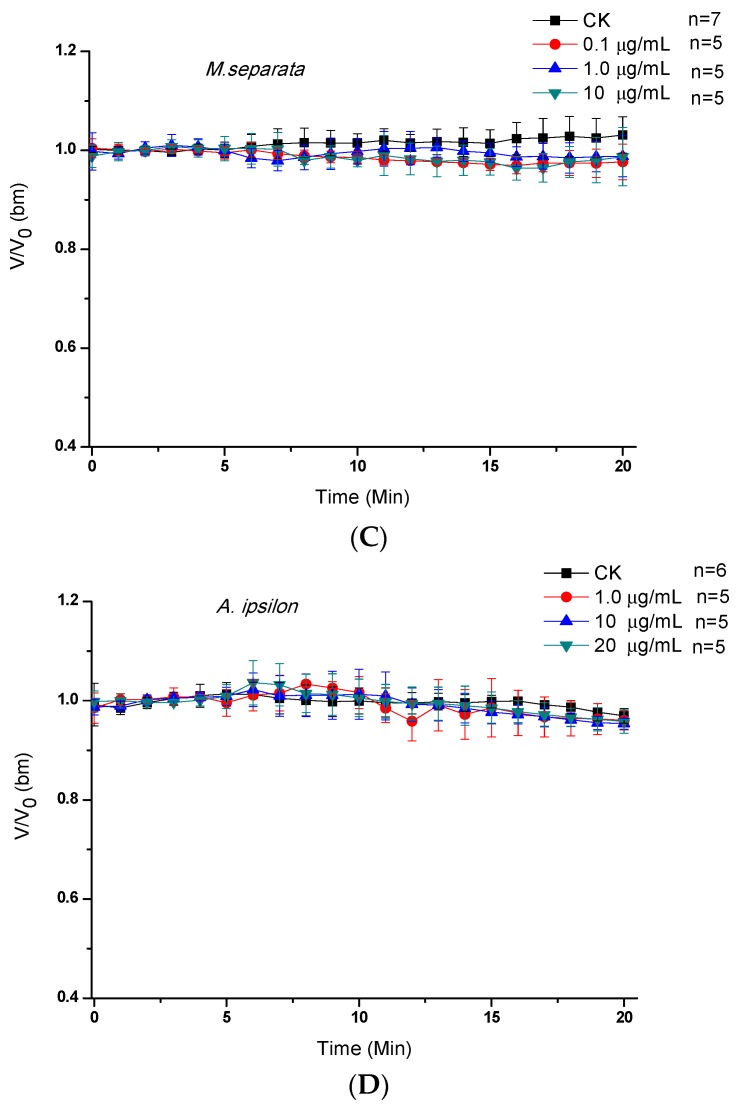
Effects of Cry1Ab on *V_am_* and *V_bm_* of midgut epithelial cells from *M. separata* and *A. ipsilon*. When the membrane potential was stable for over 5 min, 0.4 mL of the 32K solution containing activated Cry1Ab was directly added to the bath. The bath concentrations were 0.0, 0.1, 1.0, or 10.0 μg/mL. (**A**) and (**B**) are *V_am_* of *M. separata* and *A. ipsilon*, respectively; (**C**) and (**D**) are *V_bm_* of *M. separata* and *A. ipsilon*, respectively. *V* refers to *V_am_* or *V_bm_* measured at the indicated times, and *V*_0_ is the mean membrane potential measured during stabilization before the addition of toxin. Values are presented as mean ± SEM for 4 to 18 independent experiments.

The effects of Cry1Ab on *V_am_* and *V_bm_* were tested by directly adding 0.4 mL of the 32K solution containing activated Cry1Ab when the membrane potential was stable for over 5 min. The effects of the bath concentration of Cry1Ab on *V_am_* and *V_bm_* of the midgut epithelial cells from *M. separata* and *A. ipsilon* larvae are illustrated in Figure 2. The activated Cry1Ab at a dose of 0.1 μg/mL displayed negligible effects on the *V_am_* of *M. separata*. However, an obvious concentration-dependent depolarization occurred within the concentration range of 1–10 μg/mL; the PR_50_ values were reached after 14.7 ± 0.2, 9.8 ± 0.4, and 7.6 ± 0.6 min (Figure 2A). Cry1Ab (Figure 2B) demonstrated minimal effects on the *A. ipsilon* larvae even at 20 μg/mL, and the *V_am_* value only decreased by 26.3% ± 2.3% after 15 min. The concentration of Cry1Ab did not significantly affect the basolateral side of the epithelium for both larvae (Figure 2C,D).

Generally, the transmembrane electrical potential difference is 140 mV or more, which can be measured at the apical side of the columnar cells of lepidopteran larvae [29]. In the present study, normal *V_am_* was measured in the anterior region of freshly isolated *M. separata* larval midgut samples in the standard 32K solution; the results were consistent with the published values of lepidopteran larvae [23,24,25,26]. However, the *V_am_* of *A. ipsilon* was only half that of *M. separata*. The significant difference of *V_am_* may be associated with the living habits of *A. ipsilon* larvae in soil for a longer period of time as compared with most lepidopteran larvae. Furthermore, the midgut anatomy of *M. separata* is apparently different from that of *A. ipsilon*, which was relatively coarse with obvious fluff. Both insect species were fed with leaves at different pH; their midgut juice components and pH were also different [26]. These subtle distinctions can lead to the diverse membrane potentials. Therefore, the reasons for the existing differences should be further investigated and illustrated.

The effects of Cry1Ab on the *V_am_* of *M. separata* larvae were also consistent with the previously reported *in vitro* results of the *L. dispar*, *B. mori*, and *M. sexta* larval midguts [23,24]. The depolarization rate of Cry1Ab to the *M. separata* gut was similar to that of *L. dispar*. No effects were observed on both test species when the toxin concentration was 0.1 μg/mL. In the presence of 10 μg/mL Cry1Ab, the membrane potential of *L. dispar* was completely abolished after approximately 10 min [23]. However, the PR_50_ of *M. separata* was 7.6 ± 0.6 min at 10.0 μg/mL and completely lost after about 15 min. Therefore, *L. dispar* was more susceptible to Cry1Ab than *M. separata*. No significant effects on the basolateral membrane potential were noted. The activated Cry1Ab toxins bind to specific receptors and form pores in the apical membranes of the midgut epithelial cells. These pores disrupt ionic gradients and cause lysis of the epithelial cells, thereby leading to cell death [25,30]. Compared with *M. separata* larvae, the depolarized rate of Cry1Ab to the apical membranes of the midgut cells from *A. ipsilon* larvae was not significant at a dose of 20 μg/mL. These results showed that the midgut cell apical membrane of *M. separata* was at least 20-fold more sensitive to Cry1Ab than that of *A. ipsilon*. The membrane potential after force-feeding illustrated that *M. separata* was at least fivefold more sensitive than *A. ipsilon*. In addition, bioassay results showed that *M. separata* was at least eightfold more sensitive than *A. ipsilon*. The midgut cell apical membranes of lepidopteran larvae exhibit different levels of sensitivity or tolerance to Cry1A. The membrane difference is attributed to the various habits and physiological or biochemical changes, which may significantly alter the binding ability of Cry1A toxins to the midgut cell apical membranes in different lepidopteran larvae. Another set of tests showed that the *V_am_* of *M. separata* rapidly decreased in the presence of periplocoside P (PSP; unpublished work), which exerts high insecticidal toxicity against *M. separata* larvae and is insensitive to *A. ipsilon* larvae [31,32]. However, PSP showed no effect on the *V_am_* of *A. ipsilon*. Our previous study also revealed that PSP inhibits transepithelial electrolyte and fluid secretion in the Malpighian tubules of the yellow fever mosquito *Aedes aegypti* by inhibiting the V-type H^+^ ATPase [33]. PSP can significantly inhibit the total ATPase activity via a mechanism similar to the inhibition by bafilomycin, which is a known inhibitor of the V-type H^+^ ATPase. This result further indicates that the depolarization rate of the apical membrane of midgut epithelial cells responds to insecticidal activity.

According to the above results, the susceptible difference of Cry1Ab against both types of larvae in bioassay correlated with the results of force-feeding/*in vitro* apical membrane potential. The *in vitro* difference is more sensitive than the bioassay and force-feeding assay. Overall, this work provides baseline data for future research on the components relevant to the pore-forming activities of Cry toxin in *M. separata*.

## 3. Experimental Section

### 3.1. Insects

Laboratory-adapted *M. separata* and *A. ipsilon* were obtained from the Institute of Pesticide Science, Northwest A & F University (NWAFU), China. Both populations were reared under laboratory conditions (25 °C, 75% to 80% relative humidity, and a 16 h light: 8 h dark photoperiod) and were not exposed to any pesticides prior to the experiment. The *M. separata* larvae were reared on wheat leaves, and the *A. ipsilon* larvae were reared on cabbage leaves. The actively feeding sixth-instar larvae were used for electrophysiological experiments.

### 3.2. Solutions

Experiments were conducted in the standard 32K solution, which is composed of 32 mM KCl, 5 mM CaCl_2_, 5 mM MgCl_2_, 166 mM sucrose, and 5 mM Tris-HCl (pH 8.0) [27]. The solution was filtered through a 0.2 mm pore-diameter membrane (Gelman Science, Inc. , Ann Arbor, MI, USA).

### 3.3. Toxins

Purified and trypsin-activated Cry1Ab protein standard was purchased from EnviroLogix, Inc. (Portland, ME, USA). The Cry1Ab toxin was prepared from a *B. thuringiensis* strain producing the appropriate single recombinant toxins [34]. The crystal protein (inclusion bodies) was activated by trypsin and purified by rapid protein liquid chromatography with a Mono Q ion-exchange column [35]. The purified and lyophilized toxin was stored at −20 °C. Stock solutions of 2 mg/mL Cry1Ab were prepared in 25 mM Tris-HCl (pH 9.4) and stored at 4 °C. The stock solutions were diluted daily to the appropriate concentrations in the 32K solution.

### 3.4. Insect Bioassay and Treatment

Force-feeding bioassays were conducted as previously described by Abdullah *et al.* [4] with minor modifications. The selected fourth-instar *M. separata* and *A. ipsilon* larvae were starved for 24 h prior to the test. The larvae were narcotized with ether; as such, their bodies cannot freely move, but the mouthparts can still feed. Each larva was treated with 1 µL of the corresponding treatment dilution by using a pipette; the liquid was administered to the oral cavity. The larvae completely recovered within 5 min. Dilution buffer (32K solution) was fed to the control larvae. Each replicate had 25 larvae, with three replicates per treatment. Force-feeding bioassays were scored at 24 h after treatment. Lethal doses of toxin causing 50% mortality (LD_50_) were calculated by probit analysis.

To measure the membrane potential during force-feeding, the sixth-instar larvae of *M. separata* and *A. ipsilon* were fed with 1 μL of the 32K solution containing 1 and 5 μg activated Cry1Ab. The controls were only fed with 1 μL of the 32K solution.

### 3.5. Midgut Isolation

The larvae were pinned ventral side down on an ice bag, and the body wall was cut with fine microdissection scissors on the dorsal side along the longitudinal axis from the last abdominal segment to the first thoracic segment. The midgut samples were transected at each end and rinsed with the 32K solution. The peritrophic membrane with its food content and the Malpighian tubules were removed using forceps.

### 3.6. Membrane Potential Measurements

Experiments were conducted as previously described by Peyronnet *et al.* [23] with minor modifications. When the midgut is cut transversely and rinsed with the 32K solution (32 mM KCl, 5 mM CaCl_2_, 5 mM MgCl_2_, 166 mM sucrose, and 5 mM Tris-HCl; pH 8.0), both ends tend to curl back onto themselves. The bathing medium was oxygenated by the vigorous bubbling of O_2_ for 30 min prior to its use. To measure the apical-membrane potential (*V_am_*), the isolated midgut was aspirated from its posterior end into a glass pipette until its anterior end curled around the pipette tip, thus exposing the apical surface (adjacent to the lumen) of the epithelial cells. The pipette was lowered near the bottom of the testing chamber, and midgut cells were impaled with a glass microelectrode filled with 1 M KCl. To measure the basolateral membrane potential (*V_bm_*), each end of the midgut was aspirated into a holding pipette, and the epithelial cells were impaled from the basal side (facing the hemolymph). The electrode resistance was 50 MΩ to 150 MΩ. The midgut membrane potential was measured with an Axoclamp 900A (Axon Instruments, Foster City, CA, USA) and a Digidata 1440A analog-to-digital board (Axon Instruments); the pCLAMP 10.2 software (Axon Instruments) was used for data acquisition and analysis. In all experiments, the bathing solution was adjusted to maintain a potassium concentration equal to that of the 32K solution. All experiments were performed at room temperature.

The midgut was rinsed with 3 mL of the 32K bath solution *in vitro*. The membrane voltages were considered stable when *V_am_* and *V_bm_* did not change by more than 0.5 and 0.1 mV/min, respectively. After recording a stable control membrane voltage for 5 min, 0.4 mL of the 32K solution was collected with a pipette, and an equal volume of the 32K solution containing the appropriate toxin concentration was directly added to the bath. The bath concentrations used were 0.0, 0.1, 1.0, or 10.0 μg/mL. Measured voltages (*V*) were normalized relative to the voltage measured after the first 5 min (*V*_0_). Electrophysiological data are presented as mean ± SEM for more than four independent experiments. PR_50_ is defined as the time necessary to reduce the membrane potential by 50% after the toxin is added to the bath. Statistical analyses were performed with two-tailed unpaired *t-*test, and differences were considered significant at *p* ≤ 0.05.

The corresponding sixth-instar larvae of *M. separata* and *A. ipsilon* were force-fed with 1 μL of the 32K solution containing 1 and 5 μg of activated Cry1Ab. The control larvae were only fed with 1 μL of the 32K solution. After different treatment periods (0, 2, 4, 6, and 8 h), the larvae were dissected. The midgut was isolated and rinsed with 3 mL of the 32K solution. When the membrane voltage remained stable for 5 min, we recorded the membrane potential.
